# PDBe: towards reusable data delivery infrastructure at protein data bank in Europe

**DOI:** 10.1093/nar/gkx1070

**Published:** 2017-11-06

**Authors:** Saqib Mir, Younes Alhroub, Stephen Anyango, David R Armstrong, John M Berrisford, Alice R Clark, Matthew J Conroy, Jose M Dana, Mandar Deshpande, Deepti Gupta, Aleksandras Gutmanas, Pauline Haslam, Lora Mak, Abhik Mukhopadhyay, Nurul Nadzirin, Typhaine Paysan-Lafosse, David Sehnal, Sanchayita Sen, Oliver S Smart, Mihaly Varadi, Gerard J Kleywegt, Sameer Velankar

**Affiliations:** Protein Data Bank in Europe, European Molecular Biology Laboratory, European Bioinformatics Institute (EMBL-EBI), Welcome Genome Campus, Hinxton, Cambridge CB10 1SD, UK; InterPro, European Molecular Biology Laboratory, European Bioinformatics Institute (EMBL-EBI), Welcome Genome Campus, Hinxton, Cambridge CB10 1SD, UK; CEITEC - Central European Institute of Technology, Masaryk University Brno, Kamenice 5, 625 00 Brno-Bohunice, Czech Republic

## Abstract

The Protein Data Bank in Europe (PDBe, pdbe.org) is actively engaged in the deposition, annotation, remediation, enrichment and dissemination of macromolecular structure data. This paper describes new developments and improvements at PDBe addressing three challenging areas: data enrichment, data dissemination and functional reusability. New features of the PDBe Web site are discussed, including a context dependent menu providing links to raw experimental data and improved presentation of structures solved by hybrid methods. The paper also summarizes the features of the LiteMol suite, which is a set of services enabling fast and interactive 3D visualization of structures, with associated experimental maps, annotations and quality assessment information. We introduce a library of Web components which can be easily reused to port data and functionality available at PDBe to other services. We also introduce updates to the SIFTS resource which maps PDB data to other bioinformatics resources, and the PDBe REST API.

## INTRODUCTION

The Protein Data Bank (PDB, (1)) is the single global archive of experimentally determined three-dimensional (3D) structures of biological macromolecules and their complexes. The Protein Data Bank in Europe (PDBe; pdbe.org; (2)) is a founding member of the Worldwide Protein Data Bank (wwPDB; http://wwpdb.org (3)), the international consortium that manages the PDB archive. The other members of the consortium are the Research Collaboratory for Structural Bioinformatics (RCSB PDB; (4)), the Biological Magnetic Resonance Data Bank (BMRB; (5)), and Protein Data Bank Japan (PDBj; (6)). The wwPDB partners collaborate on the annotation of macromolecular structure depositions and release new data into the PDB archive each week. Since 2015, the wwPDB partners have used a unified system for deposition, curation and validation of the deposited structure data, called OneDep, with PDBe being responsible for the annotation of all depositions from European and African institutions (7). Each wwPDB partner site has developed unique services for delivering PDB data to the scientific community.

One of the principal focuses of PDBe activities is enrichment and dissemination of PDB data to the wider user community. This community not only includes domain experts such as structural biologists, but also encompasses users with varying expertise in, and knowledge of, structural data, such as bio- and chemo-informaticians, modellers, clinicians, life scientists and students. Structural biology archives are thus faced with the dual challenge of providing appropriate and consistent access to their data, as well as developing discovery and visualisation mechanisms for the benefit of all users. PDBe is addressing the following three main areas in order to meet this challenge and improve the accessibility of PDB data: enrichment of PDB data, ensuring its efficient delivery, and development of reusable Web components.

### Data enrichment

Most biological investigations require the use of multiple data resources that are often disparate and independent. Integration of PDB information with other biological resources provides biological context to the information encoded in atomic coordinates and better understanding of function and mechanism of action of biological systems. Cross-linking PDB data with other data resources also facilitates discovery of structure data. Since 2002, in collaboration with the UniProt team (8), PDBe has developed and maintained SIFTS (Structure Integration with Function, Taxonomy and Sequence, (9)), a resource providing residue-level mapping between UniProt KnowledgeBase (UniProtKB) and PDB entries, as well as annotations from IntEnz (10), GO (11), Pfam (12), InterPro (13), SCOP (14), CATH (15) and the NCBI taxonomy resources (16). Recently, PDBe has carried out a number of improvements to the way SIFTS mappings are derived and presented.

Many genes can encode for more than one protein product, e.g. through alternative splicing of mRNA. It is estimated that approximately 70% of human genes undergo alternative splicing (17) with the resulting individual protein products represented in UniProtKB as isoforms. One of the isoforms (usually the most prevalent) is termed canonical and until the recent update of the SIFTS resource, it was only this sequence to which a polypeptide chain in a PDB entry was mapped. To address this shortcoming, multiple (and potentially overlapping) mappings were enabled and, as a result, SIFTS now supports mappings to the isoform which best represents the sequence of a polypeptide chain in the PDB entry.

For example, the sequence of the isoform 3 of the N-terminal splice region of a cyclic AMP-specific phosphodiesterase from *Rattus norvergicus* (PDB entry 1LOI, pdbe.org/1loi) has no sequence identity to the N-terminus of isoform 1 (termed the ‘canonical’ sequence).

The ability to provide multiple mappings between PDB and UniProtKB also allows mappings to homologous proteins. The UniProt Reference Clusters (UniRef, (18)) provide clustered sets of sequences from UniProtKB. UniRef90 is built by clustering UniProtKB sequences with 11 or more residues such that each cluster is composed of sequences that have at least 90% sequence identity to, and 80% overlap with, the longest sequence (called seed sequence) of the cluster. If a protein chain in a PDB entry covers at least 70% of the canonical sequence in the primary mapping, alignments against all the cluster members belonging to the same set as the canonical sequence are made available from the SIFTS resource. For example, while the PDB contains structures for approximately 2800 unique human proteins, our analysis shows that a further 3300 sequences for human proteins can be mapped to structures from other organisms at >90% sequence identity.

The new version of SIFTS has also updated the rules for mapping between structures in the PDB and Pfam such that structures are now mapped to Pfam only if the entire Pfam domain is present in the sample sequence.

**Figure 1.**
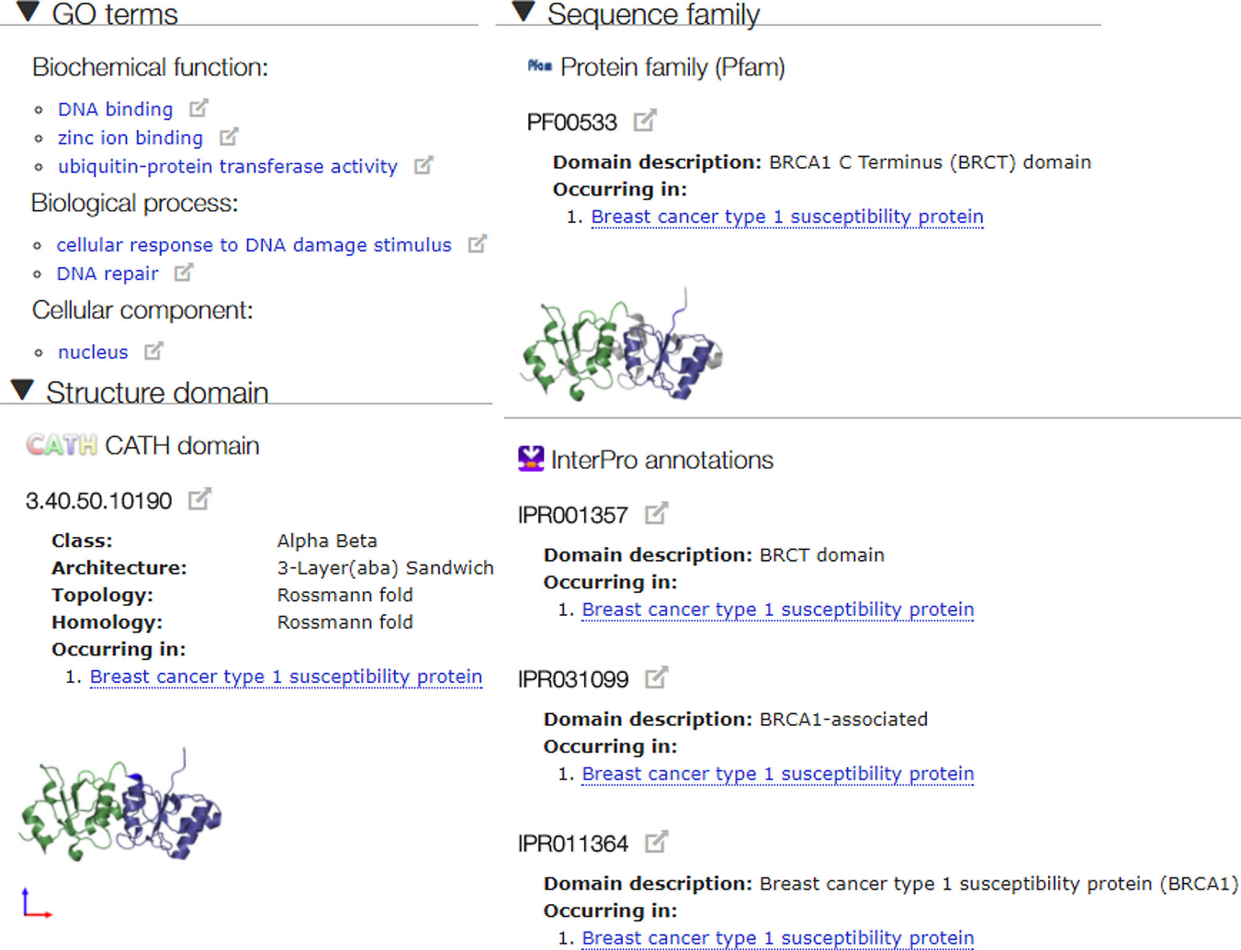
Screenshot from PDB Web pages for PDB entry 4IGK (structure of human BRCA1 BRCT in complex with ATRIP peptide, pdbe.org/4igk), showing SIFTS annotations from GO, Pfam, InterPro and CATH.

SIFTS data continues to be accessible via the PDBe REST API (pdbe.org/api) and can be downloaded from the FTP site (ftp://ftp.ebi.ac.uk/pub/databases/pdb/). We continue updating the PDB entry pages with the relevant information provided by SIFTS as shown in Figure 1.

Popular drug names are typically not provided for most ligands deposited in the PDB archive. PDBe has mapped InChIKeys (19) of all ligands in the PDB archive to those of drugs available in DrugBank (20), thus enabling searches for ligands and corresponding structures using drug names, additional synonyms, registered brand names and even E numbers used to define food additives within the European Union.

### Data delivery

Catering to a wide variety of users requires development of flexible and robust modes of access, along with presentation and visualization of macromolecular structure data. This includes logically grouping information to provide relevant context on PDBe Web pages, and developing programmatic access to the underlying data for bioinformatics use cases. In addition to accessing the structure data itself, users with different levels of expertise seek information from the PDB archive by searching for concepts they are most familiar with, such as sequences, genes, small molecules and biological functions. A search system must, therefore, support querying over a rich and extensive set of metadata and associated biological information. Lastly, the increasing size and complexity of macromolecular structure data makes real-time 3D visualization in the Web browser a challenging task.

### RESTful application programming interface (API)

Advanced data-intensive approaches are needed to connect 3D structures to the wider context of biological data and scientific literature. Many research groups develop bespoke protocols to retrieve and collate 3D structure data with other information, such as cross-links to other biological resources described above. This information often resides in multiple files, each in a different format.

As described previously (2), in order to facilitate programmatic data retrieval, PDBe has developed a public RESTful (Representational state transfer) API (pdbe.org/api), which underpins the production workflows at PDBe and provides a simple, reliable, and lightweight mechanism to query macromolecular structure data, select entries or molecules of interest, and access targeted information about them. The PDBe REST API is organised into modules pertaining to the core archive, chemistry, SIFTS, validation information and assembly information from PDBePISA (21). In the past two years, 18 additional REST call end-points were added providing a more complete coverage of underlying macromolecular structure data and metadata, while maintaining backwards compatibility. The PDBe REST API has been integrated into various external tools and services, such as the Volume Slicer (22) for Electron Microscopy Data Bank (EMDB; (23)) entries, LiteMol (Sehnal *et al.* in press), Jmol/JSmol (24) and Coot (25) to display SIFTS mappings and/or validation information, and JalView (26) to directly query PDB data from the viewer.

### CoordinateServer

Continuing advances in structure determination techniques lead to increased size and complexity of structures available from the PDB, making efficient data delivery a challenging task. Many use-cases, however, may only require a portion of the data for a PDB entry, such as a ligand and its immediate environment.

To address this challenge, PDBe, in collaboration with the Central European Institute of Technology (CEITEC, https://www.ceitec.eu/), have developed the CoordinateServer, capable of dynamically extracting and transmitting subsets of atomic coordinates for a given structure, thereby significantly reducing the network transfer size. The server can also perform common tasks, such as assembly generation, finding backbone, sidechain or ligand atoms and finding atomic coordinates of residues within a given radius from the ligand, including residues from symmetry-related molecules. The server is accessible online at www.ebi.ac.uk/pdbe/coordinates/.

### DensityServer

Data delivery challenges are even more pronounced for experimental data. Electric potential maps for models derived by electron cryo-microscopy for instance, may be several gigabytes in size. The DensityServer, also developed in partnership with CEITEC, can dynamically extract and transmit portions of experimental maps. Moreover, experimental maps can be requested as a full resolution subset (e.g., around a binding site) or as a complete map at a dynamically down-sampled resolution, enabling near instantaneous data transfer and rendering in both cases. The DensityServer is accessible online at www.ebi.ac.uk/pdbe/densities/.

The CoordinateServer can return data encoded in the PDBx/mmCIF (27) format, thus making it compatible with the data standards developed to represent structural biology information. In addition, both servers also support data compression using the new BinaryCIF format (Sehnal *et al.* in press), which uses standard PDBx/mmCIF dictionary definitions and can store macromolecular models, experimental maps, added annotations and other data. The BinaryCIF format thus, not only provides a uniform data-storage mechanism, it further reduces the size of transmitted data (Sehnal *et al.* in press), enabling even structures of large viral particles to be transferred rapidly to the user.

### Reusability—web components

Many biological data resources, including PDBe, are engaged in similar tasks in order to provide broadly similar features, such as displaying a carousel of images, formatting a search result, visualising sequences and annotations and viewing molecules in 3D (28). By utilizing Web standards such as Web components, the corresponding data and functionality can be made instantly and easily portable, thus encouraging reuse. These Web components can be shared, either to fetch third party data into a service, or to reuse the functionality to manipulate data from another resource, thereby saving development time. Furthermore, Web components ensure consistent visualization of the same data across different services, resulting in a better user experience.

Web components are reusable and customizable JavaScript-based widgets that conform to Web standards and can be freely used on Web pages in all modern Web browsers with minimal programming effort. They remove the need for technical know-how to develop interactive visualizations of data, and data can be shared across components and services without the need for replication.

PDBe has developed a library of Web components encapsulating various features. Most of them have been included in the PDBe Web site and are freely re-usable by any other Web resource by including simple custom HTML elements. The library currently contains 10 components, with more actively being developed. These include:

- **LiteMol 3D Viewer:** Integral to understanding macromolecular structure data and elucidating function is the ability to view these data in 3D. With the advent of WebGL and HTML5, a number of online tools were developed for 3D visualization of biological molecules (29,30). The LiteMol 3D Viewer (https://litemol.org, (Sehnal *et al.* in press)), developed in collaboration with CEITEC, is a new WebGL-based viewer with a low memory footprint and compatible with all major browsers without any additional plugins, and therefore compatible with tablets and mobile devices. Based on the requested visualization, LiteMol automatically queries the Coordinate and Density servers to fetch relevant atomic coordinates or portions of electron density or electric potential maps, respectively. It accepts as input PDBx/mmCIF as well as the BinaryCIF format described above. The viewer has the ability to generate interactive visualisations of 3D coordinate data with standard representations, as well as overlaid experimental data and annotations such as sequence or structure annotations and quality assessment information from wwPDB validation reports, which it dynamically queries from the PDBe REST API.
- **PDB residue interactions:** Contributed by Melis Kayikci at the MRC Laboratory of Molecular Biology (MRC-LMB, http://www2.mrc-lmb.cam.ac.uk/), Cambridge, UK, this component displays, in interactive graphical form, the atomic contacts between each of the secondary structure elements in a protein. It links directly to more detailed views on the Rajini website (http://www.mrc-lmb.cam.ac.uk/rajini). The width of the connection between each of the secondary structure elements is proportional to the number of interatomic contacts in the interface. Hovering over these connections will display the exact number of atomic contacts in that particular interaction.
- **Links to wwPDB partners:** A simple component which shows links to the three wwPDB sites providing PDB data: PDBe, PDBj and RCSB PDB.
- **PDB_REDO:** The PDB_REDO component shows the change in geometric quality and in fit to experimental data between the original PDB entry and the automatically re-refined model available from the PDB_REDO resource (31). The geometric quality score combines evaluations of the Ramachandran statistics, side-chain rotamericity and atomic packing.
- **PDB UniProt Viewer (UniPDB):** Provides a summary of PDB entries containing a sequence mapped to a particular UniProt code and displays which portion of the whole UniProt sequence is present in the PDB entry (32). The display also highlights any differences between these sequences due to, for example, engineered mutations or expression tags.
- **Experimental data:** Dynamically searches for and displays information about unprocessed experimental data related to an entry if it is found in collaborating archives (see below).
- **PDB Prints:** PDB Prints is a collection of logos in a specific order providing essential information about an entry at a glance, such as citation, taxonomy, sample production technique, experimental method and protein, nucleic acid and heterogen content (33). These logos link to detailed information about each category on PDBe Web pages.
- **PDB 3D complex:** This component gives a brief summary of the symmetry of the quaternary structure. The component also displays a confidence measure that estimates the probability of a particular quaternary structure being a biologically relevant assembly (34) and links to the PiQsi pages for further details.

**Figure 2.**
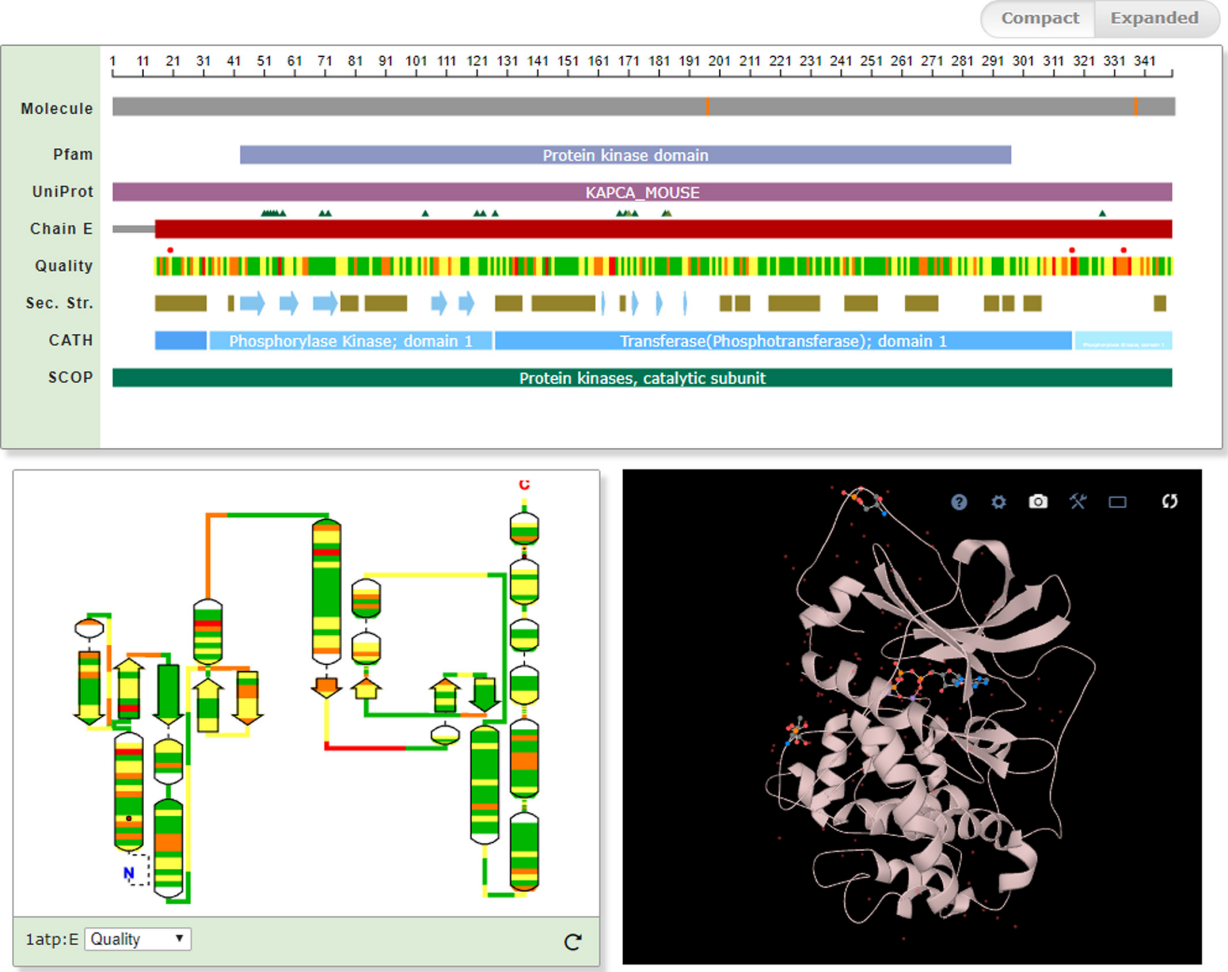
Screenshot of interactive sequence (1D), topology (2D) and 3D structure components for the catalytic subunit of cAMP-dependent protein kinase (PDB entry 1ATP, pdbe.org/1atp).

We have previously described the Sequence Feature View and the Topology Viewer (2), providing interactive linear representation of protein sequences and 2D representation of the secondary structure elements for protein chains in a PDB entry respectively, together with sequence, structure and validation annotations. Both of these viewers have now been converted into Web components. On PDBe molecule view pages, the sequence viewer, the topology viewer and LiteMol 3D viewer components work in a synchronized and interactive fashion. Selecting a residue or a secondary structure element in one of them highlights or focuses the view on the same element in the other two viewers (Figure 2).

All of the above Web components invoke the PDBe REST API, thus ensuring consistent data across the entire suite. The Web components are created using AngularJS (https://angularjs.org/), Polymer (https://www.polymer-project.org/) and D3.js (https://d3js.org/), and simple instructions how to download and reuse them are provided at www.ebi.ac.uk/pdbe/pdb-component-library. We have also incorporated most of our Web components into BioJS (35), a standard JavaScript library of over 100 open source life science related Web components.

### PDB entry pages

**Figure 3.**
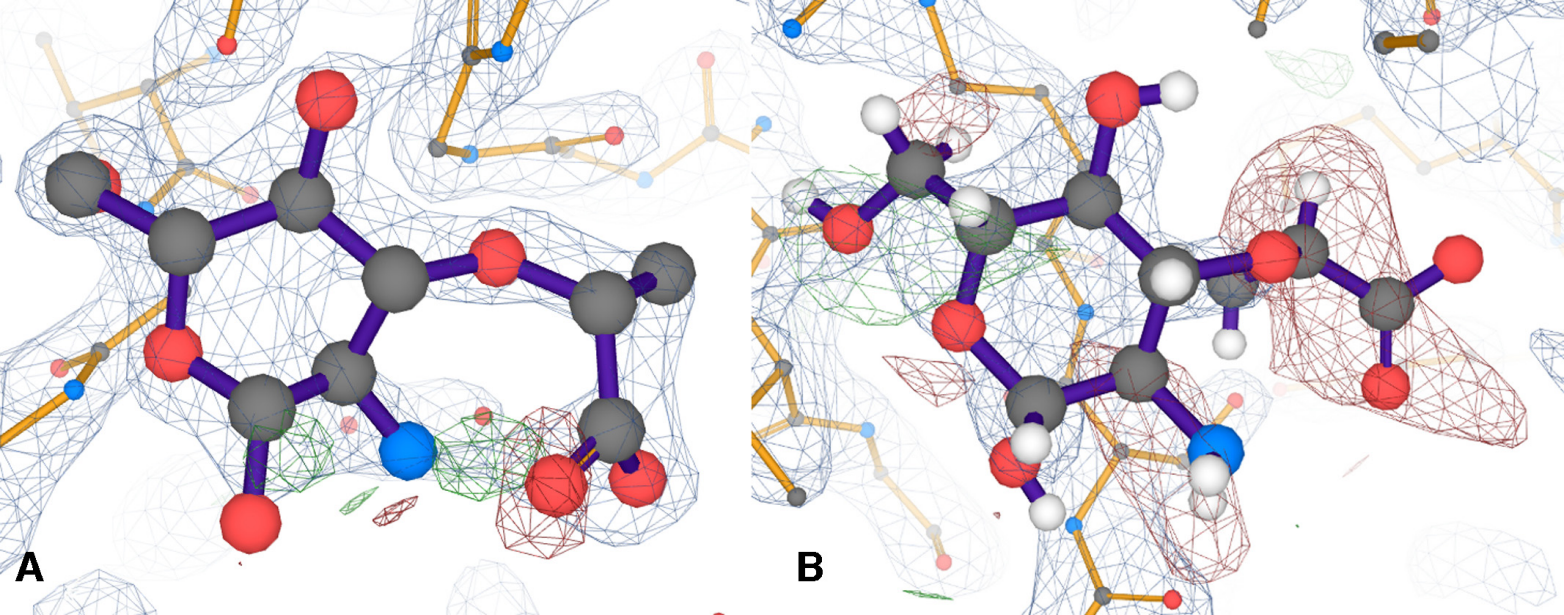
3D interactive visualisations of two instances of muramic acid in PDB structures of comparable resolution using LiteMol 3D viewer. (**A**) Data for entry 1LOD (pdbe.org/1lod) at 2.05 Å resolution. (**B**) Data for entry 5M1A (pdbe.org/5m1a) at 2.0 Å resolution. In both panels, electron density shown in blue mesh is where the experimental data and model agree (so called 2mFo-DFc map, plotted at contour level of 1.5σ, where σ is the standard deviation of the map), while electron density expected from the model and not present in the experimental data is shown in red mesh (negative values in mFo-DFc map, plotted at –3σ contour level), and electron density unexplained by the model is shown in green mesh (positive values in mFo-DFc map, plotted at +3σ contour).

The PDB entry pages serve as the main mechanism for disseminating information about PDB entries, including core PDB data, value added information and cross-references to other resources. As described previously (2), available information is arranged into six main topics, each represented by a separate Web page within the entry: summary, citations, structure analysis, function and biology, ligands and environments, and experiments and validation. A number of changes have taken place since 2016, including incorporation of the LiteMol 3D viewer capable of displaying electron density for structures determined using X-ray crystallography and electric potential maps for structures determined using electron cryo-microscopy, as shown in Figure 3.

For structures solved by multiple experimental techniques, representation of the experimental information has been improved with validation information and experimental setup described for each of the employed methods in a dedicated tab. In particular, for structures where associated small-angle scattering (SAS) data is available at the Small-Angle Scattering Biological Data Bank (SASBDB, sasbdb.org, (36)), information is retrieved directly from SASBDB via its API. The PDBe page for such entries shows key parameters derived from the scattering profile, such as the weight and oligomerisation state of the studied molecular system, and provides basic information on the sample and sample conditions, detector and radiation source.

**Figure 4.**
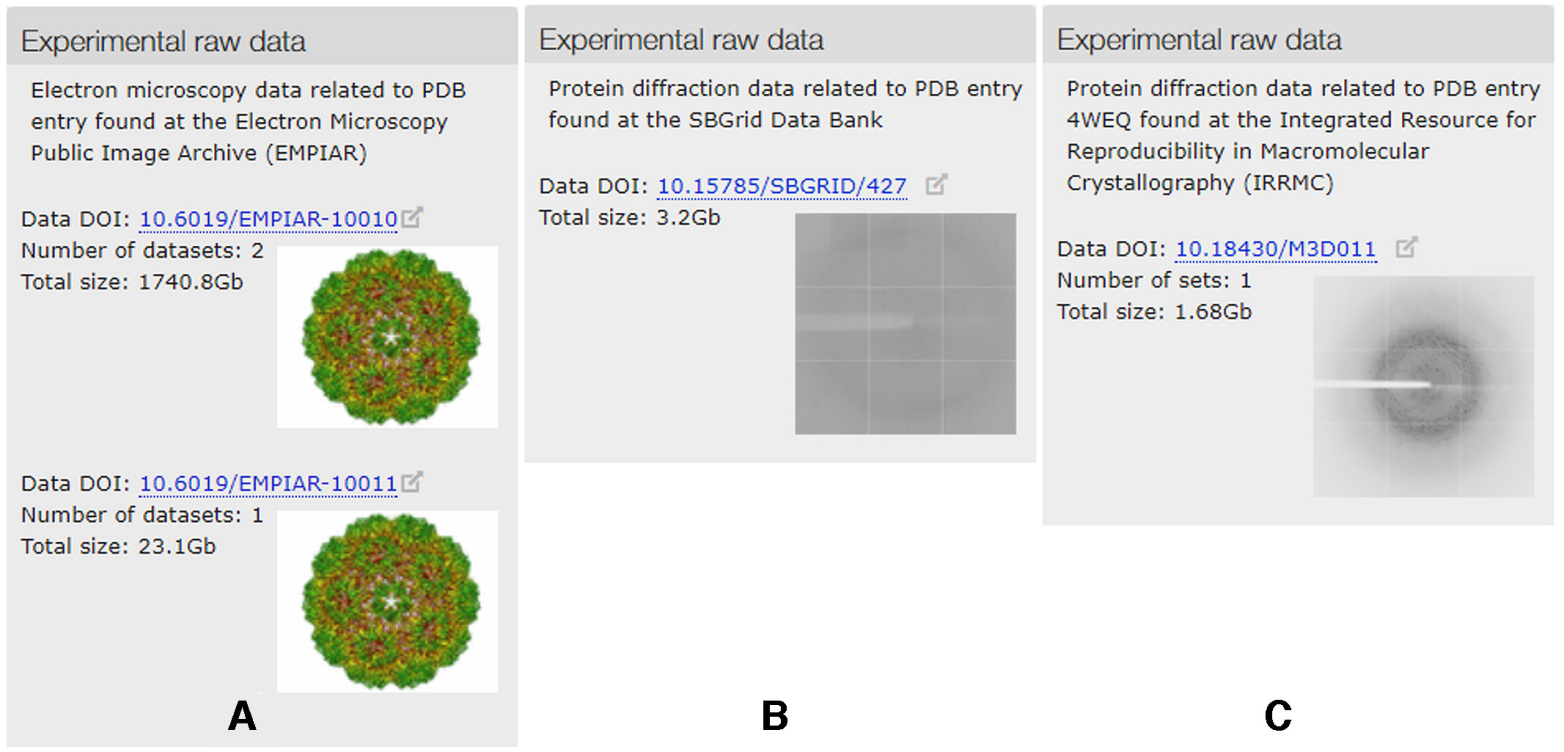
Examples showing summary and links to experimental data automatically fetched for (**A**) PDB entry 3J7L (pdbe.org/3j7l) from EMPIAR (**B**) PDB entry 5TOK (pdbe.org/5tok) from SBGrid Data Bank and (**C**) PDB entry 4WEQ (pdbe.org/4weq) from IRRMC.

PDBe entry pages have a panel that includes a navigation menu with quick links between the sub-pages of the entry and download links to all available files for an entry. It also includes context-dependent information ‘scent trail’ features in the form of Web components. For example, on the experiments and validation page, if unprocessed experimental data is available in collaborating archives, the Experimental Data Web component provides a corresponding summary and links. Currently, the widget searches three external resources for raw experimental data for an entry: EMPIAR (37), SBGrid Data Bank (38) and Integrated Resource for Reproducibility in Macromolecular Crystallography (IRRMC) (39). Examples of this are shown in Figure 4.

On the structure analysis page, links to perform structure or sequence similarity searches are presented. While viewing a particular ligand in the ligands and environments page, the ‘scent trail’ offers direct links to search for either similar ligands or sub-structures in ChEMBL (40) or binding site details for the ligand environment in PDBeMotif (41).

## FUTURE DEVELOPMENTS

We are currently working on an advanced search feature, which will allow users to query the available data by specific criteria, such as the presence of protein sequence or structural motifs, and will allow combining these criteria with logical operators. We are incorporating small molecule and binding site data into our Web pages and query mechanism, allowing users to search for structures based on binding site characteristics, as well as providing details of interactions between a ligand and its binding site on PDBe Web pages. We are also in the process of exposing ORCID (https://orcid.org/) persistent digital identifiers for entry authors, and allowing users to sign in with their ORC IDs to claim previously released entries that have no ORCID information. For the LiteMol suite, we are developing a mechanism by which users can add, save and share annotations directly in the 3D viewer. We are also continuing to develop other features of the PDBe Web site as reusable Web components, and adding more methods to the API to provide access to more data in the PDB archive files, such as richer electron cryo-microscopy metadata available in version 5 of PDBx/mmCIF.
